# Research on the Effect Mechanism of Career-Specific Parental Support Promoting Meaning in Life of Chinese Higher Vocational College Students

**DOI:** 10.3390/bs14121172

**Published:** 2024-12-06

**Authors:** Huadi Wang, Jiawen Liu, Chunyu Li, Anqi Fang, Gongjing Wang

**Affiliations:** 1 School of Education Science, Nanjing Normal University, Nanjing 210097, China; 220601023@njnu.edu.cn (H.W.); 220601024@njnu.edu.cn (J.L.); 220602088@njnu.edu.cn (C.L.); 220602089@njnu.edu.cn (A.F.); 2 School of Geography and Planning, Chizhou University, Chizhou 247000, China

**Keywords:** career-specific parental support, meaning in life, core self-evaluations, career exploration, career adaptability

## Abstract

Adolescent students’ meaning in life is an important topic of research in positive psychology and educational psychology. Meaning in life is of outstanding value to the whole career development of Chinese higher vocational college students (CHVCS), and parental behaviors have a profound impact on CHVCS’ meaning in life. In order to explore the mechanism of the role of career-specific parental support in promoting CHVCS’ meaning in life from the perspective of career, this study was based on career construction theory (CCT) and investigated 2498 CHVCS. The results showed that (1) career-specific parental support could directly and positively influence meaning in life; (2) core self-evaluations, career exploration, and career adaptability individually played a partially mediating effect in the relationship between career-specific parental support and meaning in life; (3) “core self-evaluations + career exploration”, “core self-evaluations + career adaptability”, “career exploration + career adaptability”, and “core self-evaluations + career exploration + career adaptability” play a partial chain mediating effect in the relationship between career-specific parental support and meaning in life. This study contributes to meaning in life in CHVCS, enriches career-related research based on CCT, and has important implications for career counseling from a construct perspective.

## 1. Introduction

Existential psychologist Frankl posits that human beings are inherently driven to search for meaning in life [1]. When people believe that life is meaningful, they can search for meaning and experience a sense of control over their lives. Exploring meaning in life is an eternal theme within one’s career, providing individuals with conducive conditions for growth [2]. However, today’s new generation of adolescents is growing up in an era characterized by abundant material resources and advanced information technology, which further accentuates the issue of a lack of meaning in life. The existing study indicated that Chinese higher vocational college students (CHVCS) exhibit low levels of meaning in life, manifesting as life disinterest and future ambiguity [3]. Higher vocational education is an important part of China’s modernized education system and an important supplement to general higher education, with an irreplaceable position in the cultivation of high-quality vocational and technical talents and the development of sustainable human resources. The latest data indicates that Chinese higher vocational colleges (CHVC) constitute around 49.42% of higher education institutions, with CHVCS comprising approximately 35.89%, making them a significant segment of Chinese higher education. However, CHVCS primarily emphasize technical skill development for employment, often neglecting holistic career planning for CHVCS students. This oversight results in fewer students proactively considering and preparing for their futures, leaving them ill-prepared for challenges posed by new technologies and the evolving socioeconomic landscape. Consequently, the absence of meaningful interpersonal lives (meaning in life) exacerbates the difficulties they face in career development. This study targets CHVCS students to address these issues.

Therefore, it is necessary to explore the developmental process of their meaning in life and its associated influencing factors [4]. Numerous studies in the past two decades have emphasized the plasticity and developmental characteristics of meaning in life [5], leading to a proliferation of research on its construction [6]. For instance, Cheng et al. (2011) found that the sources of meaning in life encompass five dimensions: social preoccupation, self-transcendence, personal relationships, life enjoyment, and body and mental health [7]. Similarly, Zhang et al. (2016) discovered the main sources of meaning in life among Chinese university students include self-development, social commitment, interpersonal relationships, secular pursuits, experiences in life, civilization, and autonomy, constituting seven fundamental dimensions [8]. Savickas et al. pointed out that meaning can be the result of prospective intentions as well as retrospective reflection and that people should not focus on choices in a world of uncertainty and few opportunities [9]. Instead, people should focus on the meaning of decisions by analyzing intentional processes in their life histories. From a structuralist reading, career refers to a view in motion that calls for attaching personal meanings to past memories, present experiences, and future aspirations and associating them to a pattern of life themes, and these meanings enable an individual to adapt to the social changes in their work and life [9]. Meaning-making may help individuals maintain a sense of personal agency, coherence, significance, stability, and continuity in their work lives instead of feeling passive. Career construction theory (CCT) proposes that individuals manage a variety of challenges and transitions related to their careers by imposing personal meaning on career behaviors [10].

Drawing on CCT, this study aimed to contextualize CHVCS’ career behaviors within a meaning-making perspective by illustrating relationships with career-specific parental support and meaning in life. Dietrch and Kracke (2009) defined three general facets of career-specific parental behaviors (i.e., support, interference, and lack of engagement). These include career-specific parental support in which parents encourage youths to explore their career possibilities and provide advice whenever necessary; career-specific parental interference in which parents intend to control their children’s career preparation and career aspirations by imposing their own preferences; and the lack of parental career engagement in which parents are unable or reluctant to get involved in their children’s career development [11]. Research has shown that career-specific parental behaviors significantly predicted adolescents’ career exploration, career adaptability, career self-efficacy, and career decision-making difficulties and had a significant impact on students’ career development [11,12]. Parents are not only responsible for managing their own careers but also for providing appropriate support and guidance for their children’s career development [13]. Career-specific parental support helps adolescents explore valuable information and experiences, develop a high sense of competence, and thus gain insights into future career development. Therefore, a lack of parental career-specific support may affect children’s future career resources and skill development. Incorporating a positive psychology perspective can have an impact on the development of CCT by analyzing the mixed relationship between career-specific parental support and career outcomes, such as meaning in life. Furthermore, while research on the construction of meaning in life has touched upon various aspects of social life, there has been a paucity of studies directly exploring the influence of career-specific parental behaviors on college students’ meaning in life. Most studies have situated career-specific parental behaviors within the broader context of parenting styles or family environments. However, within this broader examination, a close relationship between career-specific parental behaviors and meaning in life can be discerned.

In addition, CCT describes the mechanism of how individuals form their careers through self-construction and social construction, which defines careers from a contextual perspective, arguing that individual development is driven by adaptation to the environment rather than self-enabled career maturation. Since the cultivation of CHVC and CHVCS has obvious regional tendencies and local characteristics, most CHVC are provincial or municipal schools, and most CHVCS come from their own province or even their own city. Their relationship with their parents, a micro-environmental factor, is closer than that of general college students, and CHVCS’ career-specific parental support will inevitably affect their meaning in life, which will in turn affect the sustainable development of their careers. Therefore, based on CCT, this study explores this mechanism of influence as described in the previous section, examines to what extent the former positively affects the latter, and reveals the mechanisms behind it under a unified research framework. The study is dedicated to providing valuable insights to enhance meaning in life of CHVCS.

## 2. Literature Review and Research Hypothesis

### 2.1. Theoretical Basis

Savickas formally introduced CCT in 2002 [14], which incorporates philosophical perspectives such as constructivism and postmodernism and draws upon theoretical frameworks from other career research. The theory describes the mechanism of how individuals form their careers through self-construction and social construction and argues that careers are subjectively constructed by individuals who weave life themes to give meaning to past memories, current experiences, and future aspirations, thereby forming individuals’ entire careers. Life theme in CCT refers to the personally constructed meaning that accrues from past, present, and future experiences that shape a sense of understanding and coherence concerning the self and the world, and individuals will have a better sense of purpose. The terms “meaning”, “coherence”, and “sense of purpose” are important dimensions of meaning in life [15]. Therefore, meaning in life is closely related to CCT. The adaptive career construction model (ACCM) [14] divides the process of adaptive career construction into four components: adaptive readiness, adaptability resources, adapting responses, and adaptation results. Adaptive readiness refers to an individual’s flexibility trait or willingness to cope with career change, a stable, context-general, trait-like psychological trait. Adaptability resources refer to the individual’s ability to cope with the unfamiliarity, complexity, and uncertainty of career development tasks, career transitions, and work trauma, which is specifically embodied in career adaptability. Adapting responses refer to the way individuals respond to changes in career development tasks and work environments, including external behaviors and internal beliefs. Adaptation results refer to the degree of conformity to the requirements of the role that an individual achieves in a certain period and is the resultant expression of adaptive behaviors [16]. Theoretically, these four processes should be independent [13]. Therefore, the order and structure of this theoretical model can also be adjusted. Indeed, career development is an iterative and cyclical process, and this cycle will be characterized by a certain cyclical repetition. For example, when transitioning and switching between school to work, between jobs, and between careers, an individual will generate a cycle of adaptation, and when a new transition occurs, this cyclical cycle will be generated again. The extension of career constructs includes the whole process of individual career development, a process that includes both the adaptation of transitions in different career stages and the microcycle within a particular career stage. Duffy and Sedlacek (2010) argued that meaning in life predicts students’ career-calling development [17]. Building on previous research and the results of this study, it is evident that people with strong internal motivation and confidence in their environment would feel more meaning in life. Individuals who feel strong meaning in life are more likely to show positive attitudes and beliefs about their careers. In addition, meaning in life serves as a crucial predictor of career-calling development for students and can be regarded as an adaptive outcome of career adaptability, as explained by the ACCM of CCT [18]. However, according to self-determination theory, meaning in life as an internal belief is an important psychological resource that facilitates the emergence of behavior, and the belief that one’s life is meaningful is closely related to strong internal motivation and confidence in one’s environment [19]. The meaning in life construction model, based on theoretical research on the acquisition and maintenance of meaning in life, posits that the attainment of meaning is a complex process, involving the comparison, evaluation, and judgment of the meaning derived from situational stimuli with one’s pre-existing beliefs [20]. In the context of careers, meaning in life enables individuals to develop positive attitudes and strong beliefs about their professional paths, which is an important aspect of life. Within the framework of ACCM, meaning in life is viewed as an internal belief-shaping adaptive response.

In addition, Rudolph et al. (2019) sorted out the development of empirical research on CCT and summarized the broad applicability of CCT as a universal theory to a variety of research subjects [21]. Current research in China also shows that CCT is applicable to CHVCS. Chinese researchers have analyzed the mechanisms of the influence of perceptual social support, proactive personality, and family closeness on career adaptability and have explored the methods of intervention and enhancement of career adaptability in CHVCS by focusing on the environmental and personal factors such as school, family, and so on [22]. According to ACCM and related research [23], CHVCS experience in career development involves subjective readiness to adapt, engaging in specific career behaviors, accumulating psychosocial resources for self-regulation, and fostering stable, positive intrinsic beliefs. These factors facilitate CHVCS integration with the environment, guided by their internal beliefs. Meaning in life, reflecting positive adaptation beliefs, is integrated into this process. CCT views the nature of individual career development as a dynamic process of constructing the individual’s pursuit of mutual adaptation between the subjective self and the external environment, which explains how the individual constructs their own career development process by adopting a series of meaningful behaviors. Therefore, the original starting point of the individual’s adaptive career construction process lies in the stimulation of the external environment, and the outcome of the individual’s career development depends not only on its own factors but also on the role of environmental factors [24].

Cook et al. (2002) viewed career development from an ecological perspective, arguing that the social influences on career development can be summarized as a nested system that spreads outward from the individual as the center of the circle. The core of the system is the individual’s physical and psychological characteristics; the microsystem is factors such as family, friends, and school; the mesosystem is the interaction of the elements in the microsystem; the exosystem is factors such as the parents’ work status; the macrosystem includes factors such as values, attitudes, customs, and laws of a particular culture, and the temporal system includes social change and its impact on the factors in other systems [25]. It can be seen that the influencing factors in the process of career construction actually come from various aspects such as social and cultural backgrounds, individual emotional and mental health status, etc. Firstly, nature supports meaning in life by addressing coherence, purpose, significance/mattering, experiential appreciation of life, and place attachment [26]. Social relationships and natural connectedness provide a context for the three facets of meaning in life: organizing one’s experience, imbuing one’s life with purpose, and giving one’s life to matter [27]. Secondly, meaning in life derives from the practices that make it meaningful in the life experience of the individual, both in the past and present, and varies from culture to culture [28]. Individualist cultures emphasize independence, autonomy, and self-sufficiency, while collectivist cultures emphasize dependence, group harmony, and collective goals. Thus, the relationship between meaning-seeking and meaning-presence differs between countries with individualistic cultures and those with collectivist cultures. In collectivist cultures, the two dimensions of meaning in life seem to be mutually reinforcing, with women’s meaning in life being higher than men’s, whereas in individualist cultures, the two dimensions are mutually antagonistic, with no gender differences [29,30]. Thirdly, emotional neglect depletes the psychological resource of meaning in life and affects individuals’ understanding of their purpose and existence [31]. In contrast, positive emotions increase the likelihood of finding positive meaning by broadening thinking. For example, a grateful mindset encourages individuals to notice and appreciate the positive aspects of life, naturally serving as an incentive to find meaning in individuals’ experiences [32]. Finally, there is a bidirectional causal relationship between basic psychological needs and meaning in life, i.e., the latter is both a result of the former and an important source of promoting the former [33]. On the one hand, a good state of mental health helps individuals to better recognize and understand life, resulting in a stronger meaning in life; on the other hand, a strong meaning in life promotes the mental health of individuals and helps them to maintain a positive state of mind in the face of adversity. For example, academic stress is considered one of the greatest threats to adolescent mental health, and the effect of stressful environments on an individual’s meaning in life is a complex process, which means that academic stress may have an impact on meaning in life [34]. However, it is difficult to do an empirical analysis of the factors influencing meaning in life in the study in a comprehensive manner, and this paper only focuses on a small number of variables that have received more attention in the study and are relatively more valuable for research. The discussion of the above-mentioned factors only stays at the theoretical level.

Faced with many difficulties and uncertainties in career development, CHVCS need guidance from external resources. Among them, the family environment is one of the most important environments in the process of growing up. Though the support and encouragement of family members can have a potentially significant impact on a person’s career development, the support provided by parents cannot be easily replaced [35]. Parents provide CHVCS with different sources of meaning, for example, helping them understand their place in the world, facilitating the construction of life goals, and recognizing where they can make a difference. From the perspective of CCT, a career is a process driven by an individual’s insight into the meaning in life in terms of future career development, and intrinsic motivation plays an important role in an individual’s career development, such as career exploration and career adaptability [11]. By meeting an individual’s needs for autonomy, competence, and relatedness, situational factors can promote intrinsic motivation and proactive behaviors [36]. Career-specific parental support in this study is closely related to the realization of the needs for autonomy, competence, and relatedness in the career activities of CHVCS and thus can be taken as an important predictor of meaning in life of CHVCS who engage in adaptive career constructs. In other words, it serves as an important environmental stimulus that affects the process and outcome of CHVCS’ career constructs and thus plays a constructive role in shaping CHVCS’ meaning in life. Career-specific parental support helps CHVCS gain more insights into their future career development, generates more optimistic career attitudes, strengthens their positive personality traits, and enhances their core self-evaluations. Meanwhile, core self-evaluations can subconsciously influence CHVCS’ evaluations and perceptions of themselves and the external world. Individuals with higher core self-evaluations perceive more positive cognitions and emotions [37]. The more self-affirmation and identification they show, the more positively they experience and search for meaning in life, which means they have a higher level of sense of meaning in life [38]. When individuals have better self-evaluations, they will respond positively to problems, which is conducive to the search for meaning in life [39]. Career-specific parental support increases CHVCS’ sense of competence and enhances their motivation to engage in career exploration activities. CHVCS with high core self-evaluations have optimistic expectations for the future and can adapt more quickly to changes in their environments and be more motivated to engage in career exploration. Through career exploration, individuals can (re)construct emotions derived from interactions between personal and contextual factors by giving meaning to life experiences [40], thereby enhancing their meaning in life. At the same time, an individual’s career adaptability can largely reflect the fulfillment of their basic psychological needs, which can be inferred to the level of their meaning in life. Career-specific parental support can effectively increase the concern and curiosity of CHVCS about their careers, enhancing their self-confidence and adaptability when facing career-related problems [41] so they can better cope with career transitions and career dilemmas and adapt to the change of their career roles more quickly. Career adaptability enables individuals to expand and refine their self-concept in their professional roles and create their meaning in life, and there is thus an interaction between meaning in life and career adaptability, which in turn affects the future career development of CHVCS [42]. People with high career adaptability will purposefully construct their experiences and refresh their understanding of themselves in order to recognize meaning in their career experiences and achieve a higher level of meaningful existence [43]. At the same time, individuals with high career adaptability tend to pursue challenging career goals and invest more effort in developing skills to actively search for meaning in life [44].

**Figure 1:**
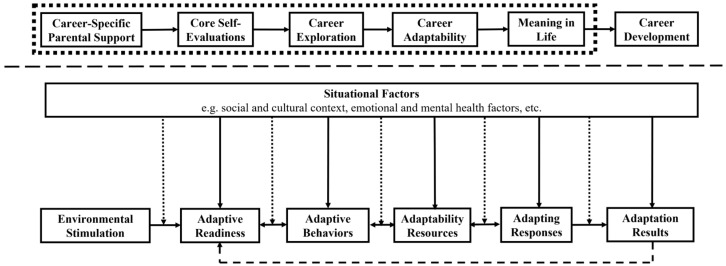
Research framework for this study. Note: Firstly, this study focuses on the linear process of the influence of career-specific parental support on meaning in life and therefore explores the cyclical and non-linear process of career development at a theoretical level only. Secondly, although the possible influence of factors such as sociocultural and mental health on career development was considered, these factors were not empirically analyzed in this study. These two points constitute the limitations of this study, i.e., this study explored the influence of certain complex factors at the theoretical level but did not specifically analyze their role in the empirical study.

In summary, the stimulation of positive environment (career-specific parental support) enhances CHVCS’ evaluation of self and external world (core self-evaluations), increases their motivation to participate in career activities (career exploration), enriches their preparation and resources for mutual adaptation of self and external world (career adaptability), and leads to changes in their internal beliefs (meaning in life), which ultimately leads to successful career development. A diagram of the relationship between the theoretical model of this study and ACCM of CCT, i.e., a diagram of the research framework of this study, is shown in Figure 1 below. However, there are some limitations in the existing research framework because this study could not attend to all aspects of the research question. First, this study only explored the relationship between career-specific parental support and CHVCS’ meaning in life from the perspective of family career environment factors, with insufficient consideration of social and cultural factors and inadequate analysis of the role of emotional and mental health. Second, the research object of this study is the specific population of CHVCS, while CCT and its ACCM do not have the attributes of such a specific population, which needs to be analyzed more cautiously in the study. Third, the inclusion of some new factors that are important in career development in this study expands ACCM but also poses a challenge to the original model, which needs to be validated by further research.

### 2.2. The Relationship Between Career-Specific Parental Support and Meaning in Life

CCT posits that the career development results of individuals hinge on individual and environmental factors [24]. The school environment (e.g., teacher support) and family environment (e.g., parental support) profoundly mold individuals’ career development [11]. Consequently, career-specific parental support, a pivotal environmental element, exerts a substantial impact on meaning in life. Meaning in life refers to “the extent to which people realize or perceive the meaning of their life, and the extent to which they perceive their purpose, mission, and primary goal in life”, which includes both “presence of meaning” and “search for meaning” [45]. Career-specific parental support means that parents encourage their children to explore their career opportunities and provide advice when necessary [11,12]. Although there is limited research exploring the relationship between career-specific parental support and individuals’ meaning in life from a career perspective, numerous studies on the impact of parental support on individuals’ meaning in life suggest a close relationship between the two constructs. Prior studies have indicated that family factors can have a greater influence on individuals’ career development than individual factors. Existing literature primarily focuses on examining the influence of early childhood and adolescent family environments or parental behaviors on individuals’ meaning in life.

Regarding early childhood family experiences, existing studies not only recognized the significant impact of early family environment and life experiences [46] but also highlighted the significant facilitative role of parental autonomy support during childhood. Building upon an examination of young adults’ evaluations of parental support during childhood and its relationship with their current meaning in life, further studies have elucidated the impact of early parental support [47]. These studies show that early family environment and parental support have a lasting impact on individuals’ meaning in life, extending beyond childhood. Regarding family experiences in adolescence, existing studies highlight the pivotal role of the family as a key source of individuals’ meaning in life [48]. Among these, family social class exerts an indirect effect through basic psychological need satisfaction [49]. Family relationships and family support, either independently or collectively, have a positive impact [50]. Family rituals through symbolic meaning and fostering of emotional interaction promote parent-child attachment [51]. Meanwhile, positive perceptions of parental support have positive implications [52]. Research on CHVCS indicates that stronger family functioning correlates with an increased likelihood of students experiencing and pursuing meaning, with parenting style affecting meaning in life via self-esteem. These studies affirm the family experiences, specifically career-specific parental support, among CHVCS. Family environment is not only an important factor influencing children’s mental health but also has a strong link with their meaning in life and suicidal intent. Career-specific parental support is a career-specific parental behavior, which is an important aspect of family education. If parents adopt more career-specific parental support, it tends to have a positive impact on CHVCS’ meaning in life. Based on the above analysis, the following hypothesis is proposed.

**Hypothesis 1.**  *Career-specific parental support has a positive impact on meaning in life among CHVCS.*

### 2.3. The Mediating Roles of Core Self-Evaluations, Career Exploration, and Career Adaptability

Core self-evaluation is an important personality trait and concept that represents an underlying and broad personality structure. It is defined as individuals’ fundamental evaluations and estimations of their self-abilities and self-worth [53]. Some studies indicated that overprotective or low levels of parental warmth can have negative effects [54] and that there are positive effects of family function [55]. Studies have found that individuals can influence their meaning in life through positive or negative self-evaluations [56]. Lower levels of core self-evaluations decrease well-being and even lead to a loss of meaning in life, while positive core self-evaluations can effectively enhance subjective well-being [57] and strengthen the experience of meaning in life. Moreover, Chinese scholars suggest that the motivational mechanism of core self-evaluations provides an explanatory basis for its positive prediction of meaning in life [53]. Based on the aforementioned research, it can be inferred that family or parental behaviors can influence individuals’ core self-evaluations, which, in turn, affects their sense of meaning in life. Therefore, the following hypothesis is proposed in this study.

**Hypothesis 2.**  *Core self-evaluations mediate the impact of career-specific parental support on meaning in life among CHVCS.*

Career exploration refers to the process in which individuals, driven by exploration motives, engage in the exploration of self and career-related environments, acquiring skills and receiving corresponding cognitive and affective feedback [58]. Family support, friend support, and teacher support can all play a positive role [59]. Specifically, parental behaviors significantly predict changes in adolescent career exploration, with adolescents experiencing low levels of career-specific parental support displaying lower levels of career exploration, and conversely, adolescents’ levels of career exploration are higher when their career-specific parental support is high [60]. Career-specific parental support directly influences college students’ career exploration and indirectly influences it through the mediating role of career self-efficacy [61]. Career development theory suggests that individuals utilize career exploration to gather information and make decisions for their future careers, thereby influencing their career development [62]. Given that career exploration is expected to facilitate individuals in better finding meaning in life, it can serve as an important mediating variable in the relationship between career-specific parental support and meaning in life. Based on the above analysis, the following hypothesis is proposed in this study.

**Hypothesis 3.**  *Career exploration mediates the impact of career-specific parental support on meaning in life among CHVCS.*

Career adaptability is regarded as the ability of individuals to cope with career role changes and maintain balance in them [63]. As an important psychological resource, it has been found to have a positive impact on meaning in life [64]. While some studies did not directly study the effect of career adaptability on meaning in life [65], they have discovered that career adaptability positively promotes calling [66], which is considered an important dimension of meaning in life [67]. In addition, studies have shown a significant correlation between the dimensions of meaning in life and career adaptability [68]. According to ACCM and the meaning paradigm, career adaptability could mediate the relationship between personality traits and meaning in life. In this view, the strengths enclosed in career adaptability could facilitate eudaimonic well-being in terms of meaning in life [18]. At the same time, whether an individual can ultimately obtain meaning in life mainly depends on the career development of the self and the environment in the process of continuous interaction, the ability to maintain a dynamic balance between the self and the environment is the individual’s career adaptability [23]. Individuals’ career adaptability reflects to a large extent the fulfillment of their basic psychological needs, thus inferring their level of meaning in life [69]. Research has also shown that career-specific parental support can affect an individual’s meaning in life, both directly and indirectly, through an individual’s career adaptability [70]. Existing studies have confirmed that career-specific parental support has a significant positive impact on adolescents’ career adaptability and its sub-dimensions [41], and similar results have been found in other studies [71]. Notably, further studies have clarified that career-specific parental support directly influences college students’ career adaptability and meaning in life, and it also indirectly influences meaning in life through the mediating role of career adaptability [70]. Accordingly, career adaptability can serve as a mediating variable in the relationship between career-specific parental support and meaning in life. Based on the above analysis, the following hypothesis is proposed in this study.

**Hypothesis 4.**  *Career adaptability mediates the impact of career-specific parental support on meaning in life among CHVCS.*

### 2.4. The Chain Mediating Roles of Core Self-Evaluations, Career Exploration, and Career Adaptability

Some studies have found that core self-evaluations predict career exploration [72] and that both variables play completely mediating roles between proactive personality and job search clarity [73]. Meanwhile, the research hypotheses in the previous part of this study have illustrated that career-specific parental support affects core self-evaluations and career exploration affects the meaning in life. Based on the above analysis, this paper proposes research hypothesis 5:

**Hypothesis 5.**  *Core self-evaluations and career exploration play a chain mediating effect in the relationship between career-specific parental support and meaning in life among CHVCS.*

Core self-evaluations have been shown to contribute to career adaptability [74]. Parental warmth directly or indirectly affects college students’ career adaptability through core self-evaluations [75]. In addition, an individual’s meaning in life is positively affected by career adaptability. Based on the above analysis, this study proposes research hypothesis 6:

**Hypothesis 6.**  *Core self-evaluations and career adaptability play a chain mediating effect in the relationship between career-specific parental support and meaning in life among CHVCS.*

Career exploration is positively associated with career adaptability and acts as a mediator between career-specific parental support and career adaptability [76]. Additionally, career-specific parental support can have an impact on career adaptability through career exploration, and the previous hypotheses in this study have identified the positive impact of career adaptability on meaning in life. Based on these findings, this study proposes the following research hypothesis 7:

**Hypothesis 7.**  *Career exploration and career adaptability play a chain mediating effect in the relationship between career-specific parental support and meaning in life among CHVCS.*

Core self-evaluations can influence job outcomes through the chain mediating effect of career exploration and career adaptability [77]. This suggests a linear impact relationship among core self-evaluations, career exploration, and career adaptability. Building upon the previous discussion on the impact of career-specific parental support on core self-evaluations and the impact of career adaptability on meaning in life, this study proposes the following research hypothesis 8:

**Hypothesis 8.**  *Core self-evaluations, career exploration, and career adaptability play a chain mediating effect in the relationship between career-specific parental support and meaning in life among CHVCS.*

**Figure 2:**
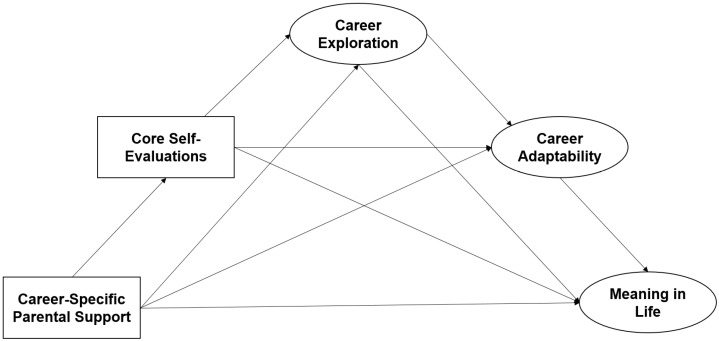
Research hypothesis model.

Based on the analysis presented above, the research hypothesis model mapped for this study is shown in Figure 2 below:

## 3. Materials and Methods

### 3.1. Participants

In this study, an online questionnaire was distributed to a random sample of CHVCS in several Chinese provinces, including Jiangsu, Guangxi, Jiangxi, Anhui, and Guangdong, for data collection. All participants were informed of the intention and requirements of the survey before completing the questionnaire used by the researchers for the study. The study was conducted by the Declaration of Helsinki (2002). The study and related documents received approval from the Ethics Committee of Nanjing Normal University. The questionnaire was administered in January 2023. A total of 3039 questionnaires were distributed, and after data screening, 2498 valid responses were retained, for an effective response rate of 82.2%. The valid sample included 794 male and 1,704 female students, with a specific distribution of 1463 students in the first year of study, 786 students in the second year, and 249 students in the third year. Among these students, 410 were from urban areas, while 2088 were from rural areas.

### 3.2. Instruments

#### 3.2.1. Career-Specific Parental Support

The Career-Specific Parental Support scale, which was the subscale of the Career-Specific Parental Behavior scale developed by Dietrich and Kracke (2009) [12], was used for measurement. The scale consists of a total of 5 items, scored using a 5-point Likert scale ranging from “1” for “completely disagreement” to “5” for “completely agreement”, with higher scores indicating a higher level of career-specific parental support. Firstly, a confirmatory factor analysis (CFA) was conducted, and the results showed that ΔCF I = 0.994, ΔTLI = 0.980, ΔRMSEA = 0.065, and SRMR = 0.011, thus meeting the criteria for scale usability. Furthermore, the internal consistency reliability of the Career-Specific Parental Support scale was found to be 0.848, thus indicating high internal consistency.

#### 3.2.2. Core Self-Evaluations

The Core Self-Evaluation scale, which was revised from the scale developed by Judge et al. (1997) [78] and adapted by Du et al. (2012) [79], was used for measurement. This is a unidimensional self-report scale consisting of 10 items. Items were scored using a 5-point Likert scale ranging from “1” for “strongly disagreement” to “5” for “strongly agreement”, with higher scores indicating higher levels of core self-evaluations. First, a CFA was conducted. The results revealed satisfactory fit indices, with ΔCFI = 0.977, ΔTLI = 0.964, ΔRMSEA = 0.054, and ΔSRMR = 0.024. Furthermore, the internal consistency reliability of the Core Self-Evaluation scale was found to be 0.840, thus indicating high internal consistency.

#### 3.2.3. Career Exploration

The Chinese version of the Career Exploration” scale, which was revised from the scale developed by Stumpf et al. (1983) [80] and adapted by Xu (2008) [81], was used for measurement. The scale consists of a total of 18 items, scored using a 5-point Likert scale ranging from “1” for “very few” to “5” for “very many”, with higher scores indicating more active career exploration. First, a CFA was conducted. The results revealed satisfactory fit indices, with ΔCFI = 0.920, ΔTLI = 0.904, ΔRMSEA = 0.070, and ΔSRMR = 0.057. Furthermore, the internal consistency reliability of the Career Exploration scale was found to be 0.954, thus indicating high internal consistency.

#### 3.2.4. Career Adaptability

The Chinese short version of the Career Adapt-Abilities Scale-Short Form (CAAS-SF) developed by Yu et al. (2020) [82] was used for measurement. The scale consists of a total of 12 items, scored using a 5-point Likert scale ranging from “1” for “not strong” to “5” for “very strong”, with higher scores indicating better career adaptability. First, a CFA was conducted. The results revealed satisfactory fit indices, with ΔCFI = 0.972, ΔTLI = 0.962, ΔRMSEA = 0.071, and ΔSRMR = 0.023. Furthermore, the internal consistency reliability of the Career Adaptability scale was found to be 0.943, thus indicating high internal consistency.

#### 3.2.5. Meaning in Life

The Chinese version of the Meaning in Life scale, which was revised from the scale developed by Steger et al. (2006) [6] and adapted by Wang et al. (2016) [83], was used for measurement. The scale consists of 10 items, scored using a 7-point Likert scale, from “1” for “completely disagreement” to “7” for “completely agreement”, with higher scores indicating a stronger meaning in life. First, a CFA was conducted. The results revealed satisfactory fit indices, with ΔCFI = 0.956, ΔTLI = 0.939, ΔRMSEA = 0.060, and ΔSRMR = 0.041. Furthermore, the internal consistency reliability of the Meaning in Life scale was found to be 0.889, thus indicating high internal consistency.

### 3.3. Analytical Methods

First, the validity and reliability of the measures, including the scales for career-specific parental support, core self-evaluations, career exploration, career adaptability, and meaning in life, were assessed through CFA and reliability testing using SPSS 27.0 and Mplus 8.3 software. Second, the Harman single-factor test and the single-factor CFA test were performed using SPSS and Mplus, respectively, to investigate the impact of common method deviation. Then descriptive statistics and correlation analysis of the variables were conducted using SPSS to gain preliminary insights into the relationships among the variables. Finally, the hypothesized model was examined to assess the significance of the mediating effects using the bootstrap method with 5000 replicate draws.

## 4. Results

### 4.1. Common Method Deviation Test

To avoid the potential interference of common method deviation, this study employed two methods. Firstly, Harman’s single-factor test was conducted by performing exploratory factor analysis on a total of 55 items representing the 5 study variables. The results indicated that 8 factors with eigenvalues greater than 1 were extracted, and the first factor accounted for less than 40% of the total variance (initial variance percentage was 36.902%) [84]. Secondly, a single-factor CFA approach was employed. A five-factor model and single-factor model were constructed separately by incorporating the variables into a structural equation model. The chi-square difference between the two models was then compared. The results showed that the chi-square value and degrees of freedom for the five-factor model were 1129.745 and 46, while the chi-square value and degrees of freedom for the single-factor model were 45,074.711 and 1430. The results of the two tests indicated that there is no common method deviation.

Afterward, career-specific parental support and core self-evaluations were also treated as latent variables, as were the other variables. The discriminant validity between the factors was verified by comparing the fit indices of the five-, four-, three-, two-, and one-factor models. As shown in Table 1, the five-factor latent variable model exhibited the best-fit indices, indicating that the five-factor model is most suitable for further analysis.

### 4.2. Descriptive Statistics and Correlation Analysis

Descriptive statistics of the data revealed that both career-specific parental support (M = 3.686, SD = 0.740) and core self-evaluations (M = 3.277, SD = 0.567) of CHVCS were at an above-average level and scored higher than the theoretical median. Career exploration among CHVCS was at an above-average level (M = 3.328, SD = 0.672). Among the four dimensions of career exploration, self-exploration had the highest mean score (M = 3.552, SD = 0.670), while the mean scores of the other three dimensions were lower than that of self-exploration and relatively close to each other. The mean scores for all four dimensions were slightly higher than moderate level. Career adaptability of CHVCS was at an above-average level (M = 3.798, SD = 0.604). Among the four dimensions of career adaptability, career confidence had the highest mean score (M = 3.853, SD = 0.677), while career concern had the lowest mean score (M = 3.730, SD = 0.692). Lastly, meaning in life of CHVCS was also at an above-average level (M = 4.972, SD = 0.833). The mean score for “search for meaning” dimension (M = 5.168, SD = 1.021) was higher than the mean score for “presence of meaning” dimension (M = 4.766, SD = 1.003). Overall, CHVCS scored slightly above the theoretical median on the five variables. However, the scores did not reach a very high level, suggesting there is still room for improvement. Particularly, attention needs to be paid to enhancing students’ meaning in life.

After conducting a correlation analysis on the data, it was observed that there were significant positive correlations (*p* < 0.01) among the five variables. Furthermore, positive correlations (*p* < 0.01) were also found among the sub-dimensions of each variable. Detailed analysis results can be found in Table 2.

### 4.3. Mediating Effect Test

First, this study modeled the mediating effect of the underlying mechanism of career-specific parental support and meaning in life. The fit indices of the model were as follows: ΔRMSEA = 0.097, ΔSRMR = 0.041, ΔCFI = 0.948, and ΔTLI = 0.926. Furthermore, career-specific parental support was found to have a direct positive effect on meaning in life among CHVCS (ES = 0.104, *p* < 0.05), with an effect size accounting for 22% of the total effect.

Second, this study examined the mediating effects of core self-evaluations, career exploration, and career adaptability. The results revealed that these three variables individually played mediating roles; “core self-evaluations + career exploration”, “core self-evaluations + career adaptability”, “career exploration + career adaptability”, and “core self-evaluations + career exploration + career adaptability” are found to have a chain mediating effect. The effect sizes for the three simple mediating effect paths were 0.061, 0.045, and 0.049, accounting for 12.9%, 9.5%, and 10.4%, respectively. The effect sizes for the four chain mediating effect paths were 0.014, 0.049, 0.114, and 0.036, accounting for 3%, 10.5%, 24.1%, and 7.6%, respectively. The total indirect effect size was calculated to be 0.368, accounting for 78%. These findings indicate that three variables mentioned above can act as mediating variables, either individually or through multiple paths involving various combinations of these variables. Please refer to Table 3 for specific results.

**Figure 3:**
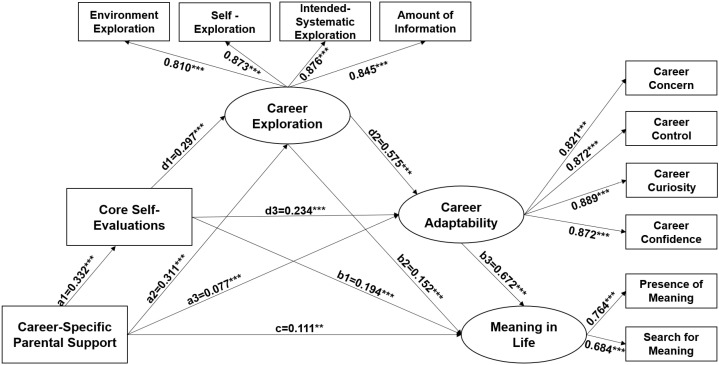
Mediating effect model. Note: ***p < 0.001, ** *p* < 0.01.

Finally, this study employed Mplus software to construct a structural equation model using the data, yielding the results depicted in Figure 3.

## 5. Discussion

CCT suggests that individuals’ development is driven by adapting to the environment and viewing career as a process of interpersonal interaction and interpretation of meaning from a constructive and contextual perspective. The theory proposes a model to explain career behaviors across the entire lifespan, offering researchers and career counselors a unique perspective to examine individuals’ career themes [85]. It also provides methods and insights for career counselors to assist visitors in making career choices, achieving career success, and enhancing job and life satisfaction [86]. This study is based on the perspective of CCT to explore the underlying mechanism through which career-specific parental support promotes meaning in life among CHVCS, making significant contributions to research on career development from a constructionist viewpoint. While many studies have demonstrated the role of parental support on meaning in life [52], few studies have explored the mediating factors involved. Career-specific parental support (e.g., instrumental assistance, career-related modeling, verbal encouragement, and emotional support) helps adolescents focus on their careers and fosters early curiosity about their career paths [87]. This study identifies the significant promotion effect of career-specific parental support on CHVCS’ meaning in life and explores its mediating factors.

### 5.1. Implications for Theories and Research

Using ACCM of CCT as a framework, the present study explores the issue of the mechanisms by which career-specific parental support promotes CHVCS’ meaning in life, aiming to gain a comprehensive understanding and appreciation of the role of positive micro-external environmental factors in facilitating the exercise of individual’s intrinsic beliefs. First, this study extends ACCM of CCT, enriches the research literature on predictors of sense of meaning in life in non-Western cultures, and deepens previous research by examining the mechanisms by which career-specific parental support promotes CHVCS’ meaning in life. Second, this study expands the understanding of career-specific parental support outcome variables. Given that this study was conducted in China in the context of collectivist culture, future research should explore whether these results can be replicated in contexts such as individualist culture. The study expands on existing theoretical perspectives on career-specific parental support and meaning in life. Given this theory and its model, Rudolph et al. (2017) used the meta-analysis method to preliminarily validate the plausibility of some parts of ACCM [88]. A large number of studies have also examined the complete sequence relationship of the four components of ACCM [89], substantiating the model. This study contributes validating ACCM in CCT, extending the relationship between career-specific parental support and meaning in life [47,50] and testing CHVCS applicability. In China, parents attach great importance to children’s careers [90]. However, due to some historical and cultural reasons, the recognition of higher vocational education is much lower than that of general higher education [91]. Limited attention has been paid to how career-specific parental support shapes meaning in life in CHVCS, and this study provides novel perspectives to enrich the domain and theory of career development in CHVCS. CHVCS are in a critical period of pursuing and discovering meaning in life during their college years [47]. Career-specific parental support, as a positive external intervening behavior, can enhance CHVCS’ core self-evaluations, career exploration, and career adaptability. This study shows that these variables are all important mediating variables in the mechanism of career-specific parental support to promote meaning in life and that career-specific parental support can enhance CHVCS’ meaning in life via these variables. When individuals have higher core self-evaluations and stronger career exploration and career adaptability, meaning in life is also enhanced. These results have important implications for enhancing CHVCS’ meaning in life.

Firstly, the study confirmed that individuals with higher levels of career-specific parental support tend to actively explore the meaning in life, leading to better career development [50]. This phenomenon can be attributed to the fact that career-specific parental support stimulates CHVCS to actively seek meaning in life during their career development process [48]. The more early parental emotional support, the more positive a child’s attitude toward the future and stronger identity commitment, both of which contributed to a greater meaning in life [47]. When early family relationships are challenging or lack the support needed for healthy identity development, meaning in life may experience some challenges [46]. Secondly, this study provided evidence for the mediating role of core self-evaluations, career exploration, and career adaptability in the relationship between career-specific parental support and meaning in life among CHVCS. Family and parental behavior affect children’s core self-evaluations [54,55], while core self-evaluations affect their meaning in life [57]. In the process of adapting to the environment, the higher the core self-evaluations of an individual, the more it also facilitates their continuous self-integration and enhances their sense of self-presence and self-determination. On the contrary, individuals with low levels of core self-evaluations show a loss of self-involvement and sense of purpose in their current lives, as well as a sense of confusion about the future and a high tendency to fall into a sense of meaninglessness [69]. The search for meaning in life begins at home, so naturally, it waits to be given by homeschooling [92]. Career-specific parental support, by addressing CHVCS’ career exploration needs, enhances their motivation and activity levels in studies and life, thereby increasing their meaningful interpersonal lives (meaning in life). Moreover, career-specific parental support positively influences CHVCS’ meaning in life through career adaptability, as it improves career adaptability by boosting self-confidence and problem-solving abilities in facing career-related challenges. This enhanced adaptability enables CHVCS to navigate career transitions more effectively and embrace unknown challenges, fostering a sense of purpose and identity. Consequently, individuals are motivated to further engage in career exploration and seek meaningful experiences in their continuous careers [70]. In summary, career-specific parental support can directly enhance CHVCS’ meaning in life and will also enhance CHVCS’ meaning in life by enhancing students’ core self-evaluations, career exploration, and career adaptability and thus meaning in life. This parental support fosters positive perceptions, encourages exploration, and enhances coping abilities in career development. Moreover, it offers valuable resources and learning opportunities for students to explore diverse career paths. Additionally, parental support acts as a buffer against negative emotions, promoting the development of career adaptability in CHVCS [61,76]. Core self-evaluations affect CHVCS’ career exploration and increase CHVCS’ meaning in life through career exploration and career adaptability. This may be due to the fact that it promotes the individual’s subjectivity and positive attribution of events, adjusts the individual’s emotions that may be present during career exploration activities, and strengthens their level of career adaptability, which can lead to a higher meaning in life. It is worth mentioning that this study found that career-specific parental support only had a higher indirect effect on meaning in life of CHVCS through career exploration and career adaptability. In other words, career-specific parental support can motivate individuals to actively participate in career exploration and gain access to more comprehensive career resources to enhance career adaptability, which in turn improves the meaning in life of self. The finding that career-specific parental support can influence meaning in life of CHVCS through multiple mediating effect paths of core self-evaluations, career exploration, and career adaptability expands the understanding of the mechanism by which career-specific parental support promotes the meaning in lives of CHVCS, which also has important practical implications for the promotion of the meaning in life of CHVCS.

### 5.2. Practical Implications

The effectiveness of career interventions based on CCT has been confirmed by previous studies [93]. The findings of this study reinforce the significance of a constructivist perspective in career counseling. Employing CCT as a guiding framework, counselors can shape strategies attuned to diverse career construction paths, effectively tackling group-specific developmental concerns. ACCM based on CCT has been the focus of empirical research by early researchers [23]. As research continues to expand, researchers have conducted career interventions for different groups, including older employees in organizations [94], new graduated nurses [95], workers with intellectual disability [96], refugees [97], adult workers with disabilities [89], and unemployed individuals [98]. In this study, the intervention population extends to CHVCS. Furthermore, in a swiftly evolving work world, the emphasis on “adaptability” is essential, with profound implications for career education and counseling practices.

Firstly, career-specific parental support can directly promote CHVCS’ meaning in life. Therefore, life education and career guidance for CHVCS require the active participation of parents. In the Chinese cultural context, interventions that promote adolescent meaning in life based on parental behavior are reasonable. CHVCS actively draws on the power of parents to promote students’ career development. School staff should strive to increase the importance that parents place on career and life planning and guide them in helping their students develop the knowledge, academic skills, and insights needed for appropriate careers [15]. Educators and career counselors can develop a parent career-related support intervention program, which could include several participatory workshops, career-related activities for children, etc. In individual counseling, counselors can have parents of student clients give their children career-specific parental support, either directly or indirectly, to help clients increase their meaning in life. Career professionals might utilize several intervention strategies targeted to increase CHVCS’ meaning in life. A narrative approach, such as the career construction interview from CCT, would serve as an effective intervention to explore and increase a client’s meaning in life. In the career construction interview, counselors ask questions, such as those relating to clients’ role models and favorite stories and sayings, and they request clients to tell short stories to reveal how they construct their identities and build careers. This interview encourages clients to engage in storytelling, through which people derive a sense of meaning and purpose in their lives and their world of work [99]. Life design intervention is another program to increase CHVCS’ ability to tell their stories and perform their identities by constructing, deconstructing, and reconstructing their career stories [100].

Secondly, this study explored the pathways through which career-specific parental support can produce a wide range of positive outcomes on meaning in life in CHVCS. In the study, it was shown that core self-evaluations, career exploration, and career adaptability enhanced the positive effect of career-specific parental support on meaning in life. By identifying and utilizing mediating variables, practitioners can more effectively design and implement interventions to achieve desired outcomes. At the same time, it is also a reminder that when assessing the effect of an intervention, one should not only focus on the final dependent variable but also gain a deeper understanding of how the independent variables have an impact through the mediating variables. By studying the role of mediating variables, researchers can more accurately understand the relationship between the independent and dependent variables and propose targeted interventions. Thus, perhaps the core mechanism for enhancing CHVCS’ meaning in life lies in promoting their core self-evaluations, career exploration, and career adaptability. Career counselors can attempt to identify the status of different CHVCS through standardized assessments based on an understanding of the antecedents of differences in meaning in life. By further understanding the distal and proximal predictors, this study provides educators and counselors with practical knowledge to enhance the meaning in life of CHVCS. First, people should pay attention to the personality development of CHVCS and focus on cultivating their career self-confidence. Parents can engage their children in career discussions and motivate them to gather vocational insights and partake in internships [90]. Second, people should pay attention to the career behaviors of CHVCS and focus on improving their behavioral positivity. Career counselors can provide parents with career-related educational training and counseling programs, yet the programs may confront several challenges [101]. Third, people should support the construction of psychological resources for CHVCS to comprehensively improve their career development abilities. Schools should host professional workshops and establish collaborative platforms for homeschool–community partnerships.

Finally, social and cultural factors and emotional and mental health are also closely linked to meaning in life, and even though they are not supported by empirical data in this study, they still cannot be ignored. Therefore, a broader range of meaning-making strategies should be explored, taking into account the above factors. First, adequate social support may provide students with more courage to explore meaning in life. Colleges can employ support group intervention [102]. Career educators and counselors can target attitudes toward a search for meaning about collectivism and individualism for intervention, as changing these attitudes may be effective for increasing individuals’ meaning in life [103]. Secondly, meaning in life can predict students’ mental health, and schools and families should strengthen humanistic care and create a favorable atmosphere to encourage and help them pursue meaning in life [104]. At the same time, CHVCS are in the important period of role-unification formation, and increasing the recognition of their own abilities and values by cultivating a growth mindset can effectively enhance their meaning in life. Third, experimental studies have shown that positive emotional manipulations can enhance meaning in life. Examples include listening to happy music, writing about happy memories or imaginary accomplishments, and reading the funnies in the newspaper [105]. In addition, many studies have proposed effective strategies for meaning-making, for example, by improving self-acceptance [106], promoting self-understanding [102], enhancing physical activity [107], using an internet-based intervention for adjustment disorder [108], and so on. Similarly, the role of school education cannot be ignored; it should incorporate meaning in life into the educational process and explore the pedagogical knowledge needed to teach the meaning in life [109]. For example, counselor-led support groups within schools should be established to address the emotional needs of students facing neglect; activities to promote meaning-making could be implemented in the school curriculum, which could include art therapy, mindfulness practices, or projects that encourage students to explore their values and aspirations [110].

### 5.3. Limitations and Future Directions

This study, despite its theoretical and practical significance, has several limitations that suggest directions for future research. Firstly, reliance on online surveys may yield invalid data, highlighting the need for paper-based questionnaires to enhance representativeness. Secondly, the focus on a specific context, CHVCS, limits generalizability, prompting exploration across diverse groups and cultural settings. Additionally, a comprehensive examination of meaning in life’s relationship with other personality traits and psychological states, like Big Five personality and resilience, is warranted. Thirdly, the cross-sectional analysis precludes causal claims. Future research should focus on substantiating the dynamic meaning-making processes through longitudinal studies and empirically evaluating the effectiveness of the proposed interventions. Fourthly, self-reported data may introduce common method biases, emphasizing the need for objective measures [111]. Lastly, limited studies on CCT exploring the mechanism of career-supportive parenting style (career-specific parental support) in promoting meaning in life in CHVCS challenge the study’s conclusions, suggesting the need for evaluating its practical value.

## Figures and Tables

**Table 1:** Comparison of different factor structure models.

Models	Factors	RMSEA	SRMR	CFI	TLI
Model1	career-specific parental support, core self-evaluations, career exploration, career adaptability, meaning in life	0.080	0.113	0.772	0.762
Model2	career-specific parental support, core self-evaluations, career exploration+career adaptability, meaning in life	0.095	0.122	0.679	0.665
Model3	career-specific parental support, core self-evaluations+career exploration+career adaptability, meaning in life	0.100	0.100	0.643	0.628
Model4	career-specific parental support+core self-evaluations+career exploration+career adaptability, meaning in life	0.105	0.105	0.605	0.589
Model5	career-specific parental support+core self-evaluations+career exploration+career adaptability+meaning in life	0.111	0.101	0.561	0.544

Note: “+” means two factors are merged into one factor.

**Table 2:** Correlation analysis among the variables.

Variables	M	SD	1	2	3	4	5	6	7	8	9	10	11	12	13	14	15
1. Career-Specific Parental Support	3.686	0.740	1														
2. Core Self-Evaluations	3.277	0.567	0.332 **	1													
3. Environment Exploration	3.206	0.801	0.347 **	0.327 **	1												
4. Self-Exploration	3.552	0.670	0.368 **	0.364 **	0.687 **	1											
5. Intended-Systematic Exploration	3.299	0.790	0.323 **	0.331 **	0.702 **	0.773 **	1										
6. Amount of Information	3.229	0.768	0.369 **	0.344 **	0.739 **	0.699 **	0.755 **	1									
7. Career Exploration	3.328	0.672	0.393 **	0.382 **	0.883 **	0.877 **	0.907 **	0.898 **	1								
8. Career Focus	3.730	0.692	0.369 **	0.403 **	0.465 **	0.644 **	0.497 **	0.499 **	0.585 **	1							
9. Career Control	3.785	0.650	0.322 **	0.483 **	0.435 **	0.603 **	0.494 **	0.473 **	0.557 **	0.716 **	1						
10. Career Curiosity	3.824	0.673	0.337 **	0.387 **	0.451 **	0.644 **	0.512 **	0.457 **	0.573 **	0.719 **	0.778 **	1					
11. Career Confidence	3.853	0.677	0.338 **	0.428 **	0.447 **	0.615 **	0.502 **	0.462 **	0.563 **	0.694 **	0.755 **	0.797 **	1				
12. Career Adaptability	3.798	0.604	0.380 **	0.473 **	0.501 **	0.698 **	0.558 **	0.527 **	0.635 **	0.873 **	0.902 **	0.916 **	0.903 **	1			
13. Presence of Meaning	5.168	1.021	0.316 **	0.301 **	0.342 **	0.535 **	0.414 **	0.380 **	0.463 **	0.586 **	0.574 **	0.616 **	0.586 **	0.658 **	1		
14. Search for Meaning	4.776	1.003	0.400 **	0.561 **	0.446 **	0.547 **	0.467 **	0.500 **	0.547 **	0.603 **	0.594 **	0.544 **	0.568 **	0.643 **	0.522 **	1	
15. Meaning in Life	4.972	0.883	0.410 **	0.492 **	0.451 **	0.620 **	0.504 **	0.504 **	0.578 **	0.681 **	0.669 **	0.666 **	0.661 **	0.745 **	0.875 **	0.870 **	1

Note: ** *p* < 0.01.

**Table 3:** Model path test results.

Name	Effect Size	Boot Standard Error	95% Confidence Interval	*p*	Percentage of Effect Size
Total Effect	0.472	0.024	[0.424, 0.521]	0.000	/
Direct Effect	0.104	0.018	[0.071, 0.139]	0.000	22.0%
Indirect Effect	0.368	0.021	[0.328, 0.411]	0.000	78.0%
Indirect Effect 1	0.061	0.008	[0.047, 0.076]	0.000	12.9%
Indirect Effect 2	0.045	0.008	[0.030, 0.063]	0.000	9.5%
Indirect Effect 3	0.049	0.014	[0.024, 0.079]	0.000	10.4%
Indirect Effect 4	0.014	0.003	[0.009, 0.020]	0.000	3.0%
Indirect Effect 5	0.049	0.006	[0.040, 0.061]	0.000	10.5%
Indirect Effect 6	0.114	0.012	[0.091, 0.140]	0.000	24.1%
Indirect Effect 7	0.036	0.004	[0.028, 0.045]	0.000	7.6%

Note: Indirect Effect 1: career-specific parental support—core self-evaluations—meaning in life. Indirect Effect 2: career-specific parental support—career exploration—meaning in life. Indirect Effect 3: career-specific parental support—career adaptability—meaning in life. Indirect Effect 4: career-specific parental support—core self-evaluations—career exploration—meaning in life. Indirect Effect 5: career-specific parental support—core self-evaluations—career adaptability—meaning in life. Indirect Effect 6: career-specific parental support—career exploration—career adaptability—meaning in life. Indirect Effect 7: career-specific parental support—core self-evaluations—career exploration—career adaptability—meaning in life.

## Data Availability

The data that support the outcomes of this study are accessible from the corresponding author. Restrictions apply to the availability of these data, which were utilized with a license for this study. Data are available from the authors with the permission of Nanjing Normal University.
